# Fecal prevalence of *Campylobacter* spp. in house dogs in Lebanon: A pilot study

**DOI:** 10.14202/vetworld.2023.2250-2255

**Published:** 2023-11-11

**Authors:** Ghassan Ghssein, Rana Barakat, Nada Nehme, Rana Awada, Hussein F. Hassan

**Affiliations:** 1 Department of Biology, Faculty of Sciences, Lebanese University, Beirut, Lebanon; 2 Department of Laboratory Sciences, Faculty of Public Health, Islamic University of Lebanon, Beirut, Lebanon; 3 Department of Environmental Sciences and Natural Resources, Faculty of Agricultural Engineering and Veterinary Medicine, Lebanese University, Dekwaneh, Mount Lebanon, Lebanon; 4 Department of Natural Sciences, School of Arts and Sciences, Lebanese American University, Beirut, Lebanon

**Keywords:** *Campylobacter*, fecal prevalence, house dogs, Lebanon, polymerase chain reaction

## Abstract

**Background and Aim:**

*Campylobacter* spp. are Gram-negative bacilli that are widely recognized as a primary cause of bacterial gastroenteritis worldwide. Campylobacteriosis is the disease caused by this pathogen. Recently, greater attention has been given to the prevalence of campylobacteriosis in different animals, including pets. These animals are considered to be significant reservoirs for this zoonosis. In Lebanon, the occurrence of *Campylobacter* infection is high. Our first-of-its-kind pilot study in Lebanon aimed to estimate the fecal prevalence of *Campylobacter* species in house dogs.

**Materials and Methods:**

Thirty-five rectal swabs were collected from male and female house dogs of different ages, both with or without diarrhea. Samples obtained from the dogs were subjected to qualitative microbiological culture testing and molecular diagnosis by polymerase chain reaction assays after bacterial DNA extraction.

**Results:**

Fecal prevalence of *Campylobacter* spp. in house dogs in this study was 17%. There was a relatively higher prevalence among young females and a significant difference between healthy dogs and those suffering from diarrhea.

**Conclusion:**

Campylobacteriosis was found to be prevalent among house dogs in Lebanon, making them potential carriers of *Campylobacter* species.

## Introduction

Worldwide, bacterial species of the *Campylobacter* genus are recognized as one of the leading causes of bacterial gastroenteritis. The prevalence of *Campylobacter* has been increasing in Lebanon, especially over the past few years [1, 2]. *Campylobacter* spp. are zoonotic bacteria commonly found in the gastrointestinal tract of humans and several species, including birds, mammals, and canines [3]. These bacteria are isolated from both symptomatic and asymptomatic individuals. Infection typically occurs after ingesting contaminated food or water. After ingestion, *Campylobacter* spp. can cause inflammation of the colon, including insinuation of mucosa with neutrophils and lymphocytes as well as a toxin-releasing inflammatory response in the host [4]. Clinical signs of infection can vary from self-limiting enteritis to bloody mucoid diarrhea, occasionally accompanied by intermittent vomiting, pyrexia, and anorexia in acute cases [1]. These bacteria can be identified by direct microscopic examination of coproculture colonies grown on *Campylobacter-*specific media or by polymerase chain reaction (PCR)-based techniques. In case of suspected campylobacteriosis, fresh feces should be collected in sterile containers and immediately refrigerated for bacterial culture [4]. *Campylobacter* are Gram-negative bacteria with a thin rod morphology (straight, curved, or spiral) distinguished based on their 16S rRNA sequences. They possess a single polar flagellum and can have a flagellum at each pole, which gives *Campylobacter* a distinctive high mobility. This feature allows easy identification and diagnosis when observed under a microscope. *Campylobacter* spp. are described as fastidious bacteria due to their relatively limited ability to utilize available nutrients and their slow growth rate compared to other intestinal bacteria [5]. *Campylobacter* spp. are mesophilic germs that grow at 37°C or 42°C). Among the thermotolerant *Campylobacter* species with the ability to grow and develop at 42°C, three species are of major interest in public health and are most frequently encountered in canine pathology: *Campylobacter jejuni, Campylobacter coli*, and *Campylobacter upsaliensis* [6].

The prevalence of *Campylobacter* spp. in dogs depends on several factors, including age, geographic region, season, environment, and the quality of provided food and water [4, 7]. In house dogs, the transmission of *Campylobacter* spp. can occur through either direct or indirect fecal-oral route [1]. Fluoroquinolones, tetracyclines, macrolides, and aminoglycosides are frequently used to treat this infection in animals and humans [8]. Human campylobacteriosis arising from food sources has been extensively studied, whereas environmental risk factors such as exposure to fecal material from animals have been understudied [9]. A previous study by Moreira Lima *et al*. [10] on birds have identified the link between campylobacteriosis and environmental fecal contamination.

In household dogs in Lebanon, *Campylobacter* remains an underdiagnosed pathology or overlooked as merely antibiotic-responsive acute enteritis. Therefore, this pilot study aims to estimate the fecal prevalence of *Campylobacter* spp. in house dogs in Lebanon using two different tools.

## Materials and Methods

### Ethical approval

The study was approved by the Institutional Review Board at our university, and the corresponding IRB approval number was LAU.SAS.HH1.4/2019. Throughout the study, a licensed veterinarian was present with a comprehensive questionnaire that was completed for each animal.

### Study period and location

This study was conducted with respect to animal welfare from April to September 2019. Fecal swab samples were collected from 35 house dogs at a veterinary hospital in Mount Lebanon for this pilot study. The samples were processed at the Lebanese Agricultural Research Institute.

### Sample collection

All dogs included in the study were on a dry food diet and had received complete vaccinations. Samples were collected from both males (19 dogs, 54%), and females (16 dogs, 46%), encompassing different ages: 15 dogs were below 1 year old (43%), and 20 dogs were above 1 year old (57%). In addition, different symptom manifestations were observed, with seven dogs suffering from diarrhea (20%) and 28 dogs being asymptomatic (80%).

To collect the samples, sterile Transwabs were used, and 5 mL of buffer was added to each tube before being transported directly to the microbiology laboratory at the Lebanese Agricultural Research Institute (LARI). No animals were harmed during the sample collection.

### Media and bacterial culture preparation

Modified charcoal cefoperazone deoxycholate agar (mCCDA) base media were prepared according to the manufacturer’s instructions (HiMedia, Mumbai, India). Briefly, to prepare the media, 22.4 g of powder was suspended in 1 L of distilled water and brought to boil until dissolved. The mixture was then sterilized in an autoclave and allowed to cool down to 50°C. One vial of the *Campylobacter* CCDA selective supplement, containing cefoperazone and amphotericin B antibiotics, was added to the cooled media and carefully mixed. Using the direct plating technique, each sample was swabbed on a dried Petri dish containing the mCCDA media using the four quadrants method. The dishes were then incubated at 37°C for 48 h under microaerophilic conditions using atmospheric generators (GENbox Microaer, bioMérieux, France).

### Identification of *Campylobacter* spp

Suspected typical light-gray colonies were examined under a microscope after the Gram staining. Colonies containing “S”-shaped or spiral bacteria with darting motility were identified as *Campylobacter* spp. They were then isolated and re-cultured on a fresh Petri dish containing mCCDA for isolation. The newly prepared dishes were incubated for 48 h under the same conditions (microaerophilic atmosphere at 37°C). The colonies were subsequently re-examined under a microscope to confirm the presence of *Campylobacter* spp.

### Bacterial DNA extraction

For bacterial DNA extraction, the Quick-DNA™ Faecal/Soil Microbe Miniprep Kit (ZYMO Research, Irvine, CA, USA) was used. DNA was extracted using 150 mL of samples following the manufacturer’s instructions. The samples were directly added to a ZR BashingBead™ Lysis Tube (ZYMO Research, Irvine, CA, USA) and efficiently lysed using a bead beater homogenizer. This technique does not require the use of any organic denaturants or proteinases that could harm the DNA. Zymo-Spin™ Technology (ZYMO Research) was then applied to isolate the DNA, which was subsequently filtered to remove any substances that might inhibit the PCR procedure, such as humic acids and polyphenols. The purified DNA was stored at −20°C and used for PCR within a week, following DNA quantification using a nanodrop (BioSpec-nano Micro-Volume Spectrophotometer by Shimadzu, Columbia, Maryland, USA).

### Campylobacter detection using PCR method

Polymerase chain reaction is a method used in molecular biology to detect DNA sequence(s) of different pathogenic agents, including *Campylobacter* spp., by allowing the amplification of a targeted DNA sequence using specific primers. The primers used in this study targeted the 16S rRNA gene, which is specific to *Campylobacte*r spp., and had the following sequence:

Forward primer: 5’-GGAGGCAGCAGTAGGGAATA- 3’
Reverse primer: 5’-TGACGGGCGGTGAGTACAAG- 3’

Using MasterMix (Biomérieux), the amplification reactions were performed in a mixture containing water, Taq buffer, deoxynucleotide triphosphate (dNTP), Taq polymerase (Taq PCR Core Kit, Qiagen), and primers. Amplification reactions were performed using a DNA thermal cycler (Veriti 96 Well Thermal Cycler, Applied Biosystems, Thermo Fisher Scientific, Waltham, Massachusetts, USA)). The amplification generated 1044 bp DNA fragments corresponding to the *Campylobacter* genus. The amplified products were identified by electrophoresis in a 1% (weight/volume) agarose gel in TBE buffer 1× (GIBCO® by Life Technologies™, Waltham, Massachusetts, USA) along with a 1 Kb Plus ladder (ThermoScientific GeneRuler 100 bp DNA Ladder, Lithuania, European Union). Subsequently, the gel was stained with ethidium bromide and exposed to ultraviolet (UV) light using UVP (GelDoc-it™ Imaging System, Bio-Rad, Hercules, California, USA).

### Statistical analysis

Statistical analyses were conducted using the statistical package for the social sciences software version 24.0 (IBM Corp., NY, USA). This software was used as well for data management and cleaning. Descriptive statistics were performed and reported as frequencies and percentages for categorical variables. The Chi-square test was used to assess significant differences between the categorical variables. The significance level was set at p < 0.05 for all statistical analyses.

## Results

### Fecal culture results

Following the direct fecal culture on mCCDA media and 48 h of incubation, the typical colonies, distinguished by their small gray point shape, were examined and verified as *Campylobacter* under an optical microscope following Gram staining. The colonies were then isolated and cultured in peptone water for maximum proliferation. After incubation, pure cultures of *Campylobacter* were obtained.

#### Fecoprevalence of Campylobacter spp. in dogs according to age, sex, and symptoms

**Figure-1:**
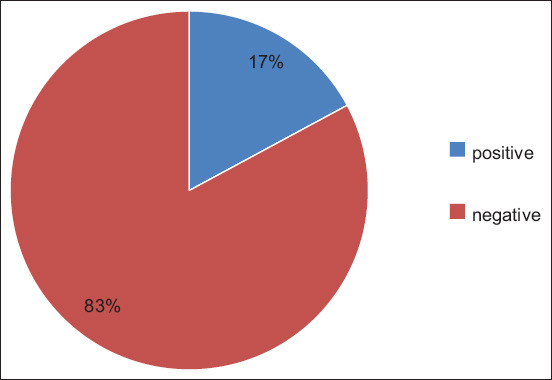
Percentage of positive and negative samples.

**Figure-2:**
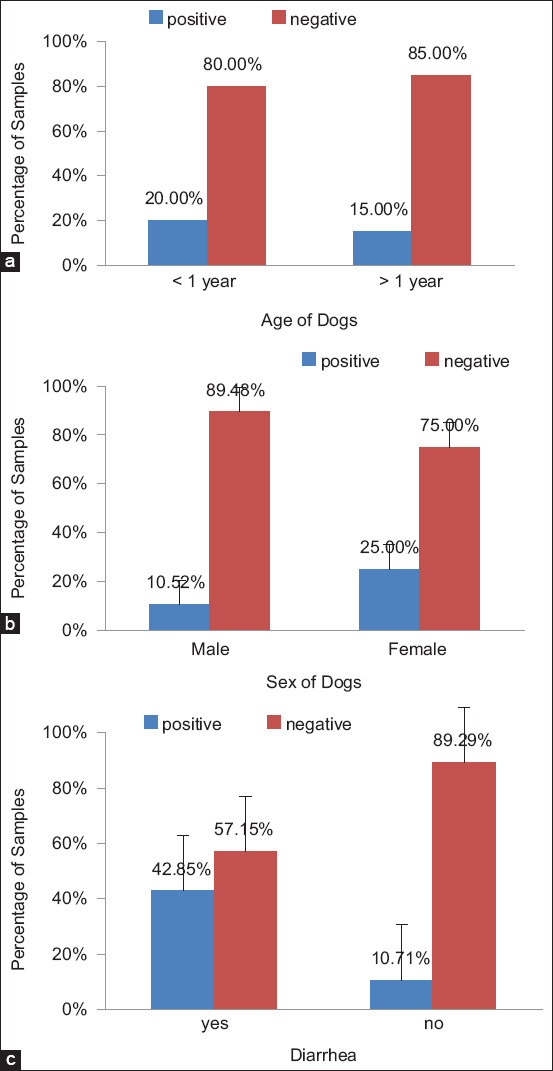
Percentage of positive and negative samples according to (a) age, (b) sex of dogs, and (c) symptom.

Out of the 35 dog fecal samples tested in the present study, *Campylobacter* spp. were isolated from six samples, reflecting a prevalence of 17% (Figure-1). Figure-2a shows the *Campylobacter* rate according to age. Among dogs below 1 year of age, 20% of samples were positive, while among those above 1 year of age, 15% of samples were positive. Figure-2b shows the *Campylobacter* rate according to gender. For male dogs, 11% of samples tested positive, while 25% of female dogs tested positive. In other words, the prevalence of *Campylobacter* spp. was higher among female dogs below 1 year of age. Figure-2c shows that 43% of dogs suffering from diarrhea were confirmed positive. On the other hand, 90% of healthy dogs (with no occurrence of diarrhea) tested negative for campylobacteriosis. Table-1 shows the statistical differences in *Campylobacter* prevalence in dogs according to gender, age, and the presence of diarrhea symptoms. The results showed a significant association between symptoms and test results (p < 0.05), while the differences in terms of gender and age were not significant.

**Table-1:** Statistical significance of *Campylobacter* spp. in dogs according to gender, age, and presence of diarrhea.

Independent variable	Prevalence of *Campylobacter* spp. n (%)	p-value
Gender		
Male (n = 19)	2 (11%)	0.583
Female (n = 16)	4 (25%)	
Age		
Below 1 year (n = 15)	3 (20%)	0.258
Above 1 year (n = 20)	4 (15%)	
Diarrhea		
Yes (n = 07)	3 (43%)	0.044
No (n = 28)	3 (11%)	

### Polymerase chain reaction results

**Figure-3:**
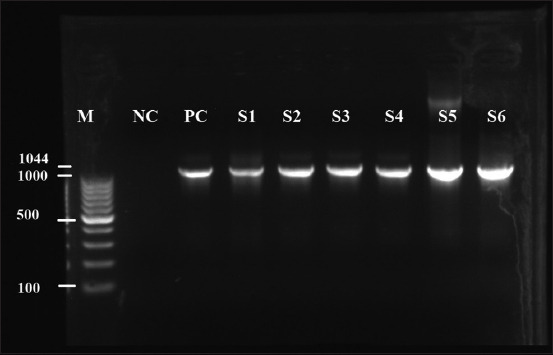
Polymerase chain reaction results on agarose gel (M=Marker, PC=Positive control, NC=Negative control, S=Sample).

*Campylobacter* is a bacterium that could be present in samples without being detected by culture. Under unfavorable growth conditions, these microorganisms may form viable but non-culturable cells [11]. Bacterial death prevents their detection under a microscope, even if they are able to proliferate after incubation. To avoid false negative results by bacterial culture, PCR was performed for the detection of bacterial DNA. The fecal culture results yielded positive for 6 out of 35 dogs (17%) for the prevalence of campylobacteriosis in house dogs. This result was confirmed by PCR, which also yielded same positive results for these six dogs (Figure-3). A molecular weight marker was used to assess all parameters. The sample with a molecular weight equal to the expected PCR product size 1044 bp was judged to be positive.

## Discussion

Most studies on pathogenesis and prevalence of campylobacteriosis in Lebanon have focused mainly on humans and poultry [2, 12, 13]. No studies have focused on the prevalence of *Campylobacter* spp. in the canine population. For the first time in Lebanon, our study revealed that a fecal prevalence of *Campylobacter* spp. at 17% in a sample of house dogs, especially among young female dogs (below 1 year old) suffering from diarrhea (Table-1). According to the a previous study by Torkan *et al*. [5], the fecal prevalence of *Campylobacter* spp. in dogs was 6%, 16%, and 28% in Austria, Iran, and India, respectively. Higher rates of *Campylobacter* spp. isolation were recorded in male dogs than in female dogs, but the difference was not statistically significant [5].

Immature dogs (below 1 year old) had a higher prevalence of campylobacteriosis in comparison to adult dogs. A combination of many risk factors represents the most possible cause of campylobacteriosis in puppies, including weakened immune system and the absence of a previous exposure to these bacteria [14].

In fact, all age groups can be carriers of *Campylobacter*. spp. due to several factors, such as coexistence of gastrointestinal diseases, weakened immunity, environmental sanitary measures, and the diet of dogs. However, other studies have shown that the prevalence of *Campylobacter*. spp. in adult dogs (above 1 year of age) was significantly higher than in dogs aged below 1 year [15].

Diarrhea is a controversial risk factor for campylobacteriosis in dogs [15]. Based on our results, the prevalence of *Campylobacter* spp. was higher in dogs suffering from diarrhea in comparison to healthy dogs. In contrast, the previous study [16] found no difference in the prevalence of *Campylobacter* spp. between diarrheic and non-diarrheic animals, emphasizing subclinical campylobacteriosis, questioning the specific pathogenicity of these bacteria in companion animals. Dogs living in kennels or shelters have an increased prevalence of infection compared to house dogs [16].

The One Health approach for campylobacteriosis is a comprehensive and multidisciplinary strategy that recognizes the interconnections between human health, animal health, and the environment in preventing and controlling the disease. By addressing the issue from different angles, the One Health approach aims to mitigate the transmission of Campylobacter from animals to humans and reduce the burden of campylobacteriosis on both public health and the agricultural sector. Some key aspects of the One Health approach for campylobacteriosis [17, 18]:

1. Surveillance and monitoring: Includes in-depth monitoring of Campylobacter prevalence in both human and animal populations to identify potential sources of transmission and outbreak trends, and integration of data from human surveillance systems and veterinary networks to comprehensively understand the disease dynamics.
2. Epidemiological Research: Involves conducting research to identify risk factors associated with the transmission of Campylobacter between animals and humans and investigating the transmission pathways and reservoirs to develop effective preventive measures.
3. Antimicrobial Resistance Management: Includes promoting the responsible use of antibiotics in both human medicine and animal agriculture to reduce the emergence and spread of antimicrobial resistance in Campylobacter, and studying the mechanisms of resistance and identifying strategies to combat antimicrobial-resistant strains.
4. Food Safety and Hygiene Practices: Involves educating the public, food handlers, and farmers about proper food safety and hygiene practices to minimize the risk of Campylobacter contamination in food products, and implementing good agricultural practices to reduce Campylobacter prevalence in the food production chain.
5. Environmental Management: Includes studying the environmental factors that contribute to the persistence and transmission of Campylobacter in the environment, and implementing measures to prevent environmental contamination, especially in water sources used for agriculture and human consumption [17, 18].

## Conclusion

*Campylobacter* is the leading cause of acute gastroenteritis in humans and some animal species. Many risk factors contribute to the prevalence of campylobacteriosis, including hygiene, contact with contaminated animals, and the quality of provided food and water. Our study is the first-of-its-kind pilot study to estimate the prevalence of *Campylobacter* spp. in house dogs in Lebanon. It has been reported that dogs, especially diarrheic young females, are potential carriers of *Campylobacter* spp. with a prevalence rate of 17%. Control methods should be implemented to decrease or even prevent campylobacteriosis in house dogs and thus, prevent its transmission to humans. In this context, both pet owners and veterinary practitioners play a critical role in reducing the incidence and transmission of this disease. Practices include adhering to hygienic procedures, educating people about decontamination of food and water, and incorporating diagnostic testing for *Campylobacter* into routine veterinary work, especially when dogs suffer from gastrointestinal disorders. In addition, extensive and larger research projects are required to understand the epidemiology and the antimicrobial resistance of *Campylobacter* spp. in Lebanon, thus improving the general health of humans and animals.

## Authors’ Contributions

HFH, GG, NN, and RA: Designed the study and drafted the manuscript. RB and GG: Collected the samples and did the laboratory work. All authors have read, reviewed, and approved the final manuscript.
